# Age-related changes in the gut microbiota of a long-lived seabird suggest divergence from mammalian models

**DOI:** 10.1098/rsos.252018

**Published:** 2026-09-09

**Authors:** Eveliina Hanski, Kieran Bates, Robert Hughes, Mark Newell, Alex Penn, Aura Raulo, Liz Scott, Liping Tang, Annette L. Fayet

**Affiliations:** ^1^ Collegium Helveticum, ETH Zurich, Zurich, Switzerland; ^2^ Department of Biology, University of Oxford, Oxford, UK; ^3^ Queen Mary University of London, London, UK; ^4^ Royal Society for the Protection of Birds, Sandy, UK; ^5^ UK Centre for Ecology and Hydrology, Wallingford, UK; ^6^ Fair Isle Bird Observatory, Shetland, UK; ^7^ Department of Computing, University of Turku, Turku, Finland; ^8^ Shiants Seabird Research Group, Shiant Isles, UK; ^9^ Institute of Zoology, Chinese Academy of Sciences, Chaoyang, People's Republic of China; ^10^ Norwegian Institute for Nature Research, Trondheim, Norway

**Keywords:** microbiota, age, seabird, Atlantic puffin

## Abstract

Gut microbiota plays key roles in shaping host development, immunity and physiology. The assembly of vertebrate gut microbiota during early life follows relatively predictable trajectories across several mammalian species, largely driven by maternal transmission routes. However, birds lack these transmission mechanisms owing to their oviparity, potentially leading to different age-related microbiota patterns. Here, we investigate gut microbiota variation in Atlantic puffin (*Fratercula arctica*) chicks and adults across six British populations spanning distinct marine regions using 16S rRNA amplicon sequencing. We find that chick microbiotas show strong population-level structuring that disappears in adults, with spatial patterns particularly pronounced in anaerobic versus aerotolerant components of the microbiota. Puffin microbiotas become increasingly individualized and aerotolerant with age, while taxonomic richness remains stable—patterns that contrast with the convergent, richness-increasing trajectories observed in several mammalian species. Our results suggest that environmental exposure is a key driver of early-life microbiota assembly in birds, highlighting the value of avian systems for understanding vertebrate microbiota development beyond mammals.

## 1. Introduction

The vertebrate gut microbiota plays essential roles in shaping host physiology, development and immune function [1–3]. At the same time, gut microbial communities are highly dynamic, varying across hosts, populations, life stages and environments [4–10]. Understanding the ecological and developmental drivers of this variation is key to linking microbiota structure with function in natural systems.

Most insights into gut microbiota dynamics come from studies in humans and laboratory animals [1,11–13], with wild mammals receiving increased attention in recent years [14–19]. Despite birds accounting for over 5000 more species than mammals [20], avian gut microbiota remains considerably less studied. Crucially, as in mammals, microbial communities of the avian gut have been linked to host health, fitness and behaviour, representing both key biological processes and central research themes in ecology to conservation [4,5,10,21–23].

Early life represents a critical window for microbiota establishment, with lasting consequences for host development and health [1,24,25]. For the majority of mammals, microbial seeding begins at birth when the previously sterile gut is colonized during passage through the birth canal [26–28], and continues via maternal transfer (largely through lactation) and environmental acquisition [12,29]. These tightly regulated exposure routes are thought to contribute to broadly conserved age-related microbiota trajectories across several mammalian species, including increasing richness and evenness during early development [12,14,17,30–32] (but see [33]). Birds, however, follow fundamentally different patterns owing to their distinct reproductive biology and early-life environments. Birds are oviparous and lack these maternal microbiota transmission routes. Instead, early-life microbial acquisition is probably shaped by the environment (e.g. soil, nest materials) and social interactions such as feeding (parent to offspring). These more variable transmission routes may lead to less predictable microbiota trajectories during early development. This hypothesis is supported by studies showing variable age-related changes in alpha diversity across bird species [21–23,34,35] (in contrast, richness and evenness generally increase during early development among mammals [12,14,17,30–32] (but see [33]). These fundamental differences in reproductive biology mean that mammalian findings cannot be directly extrapolated to birds, positioning avian species as essential comparative models for understanding microbiota assembly across vertebrates.

In this study, we investigate the gut microbiota of the Atlantic puffin (*Fratercula arctica*), a long-lived (average lifespan approx. 25 years [36]), migratory seabird with ecology that makes it particularly suited for studying how age and environment shape the gut microbiota beyond mammalian hosts. Specifically, chicks develop in underground burrows, where they probably acquire microbes from soil and nest environments. They are also provisioned with a relatively narrow range of marine prey items carried in their parents’ beaks [37], and are thus dosed with microbes from both parents and their diet. By contrast, adults forage widely at sea and migrate between breeding seasons, potentially encountering common environmental microbial sources across populations [38]. Puffins are also of particular research interest as they are increasingly vulnerable to environmental change, with population declines across their North Atlantic range leading to their IUCN listing as ‘vulnerable’ [20] and inclusion on BirdLife International's European Red List of Birds (2021) [39]. Furthermore, their overlapping diet and breeding habitats with several other North Atlantic seabird species make insights from puffin microbiota potentially applicable to understanding gut microbial ecology across a broader range of seabirds. We used 16S rRNA amplicon sequencing of faecal samples collected from chicks and adults across six British populations spanning ecologically distinct marine areas (Celtic Sea, Hebrides, Orkney, Shetland, Firth of Forth). We examined how age and geography influence gut microbiota diversity, composition and oxygen tolerance as measures of microbiota structure and function. Partitioning the microbiota by bacterial aerotolerance has recently been applied to investigate social transmission routes in a cooperatively breeding passerine, revealing that anaerobic and aerotolerant communities respond differently to the closeness of social interactions [40], supporting the utility of this approach for disentangling ecological drivers of microbiota variation in wild birds.

## 2. Methods

### 2.1. Sample collection

**Figure 1.**
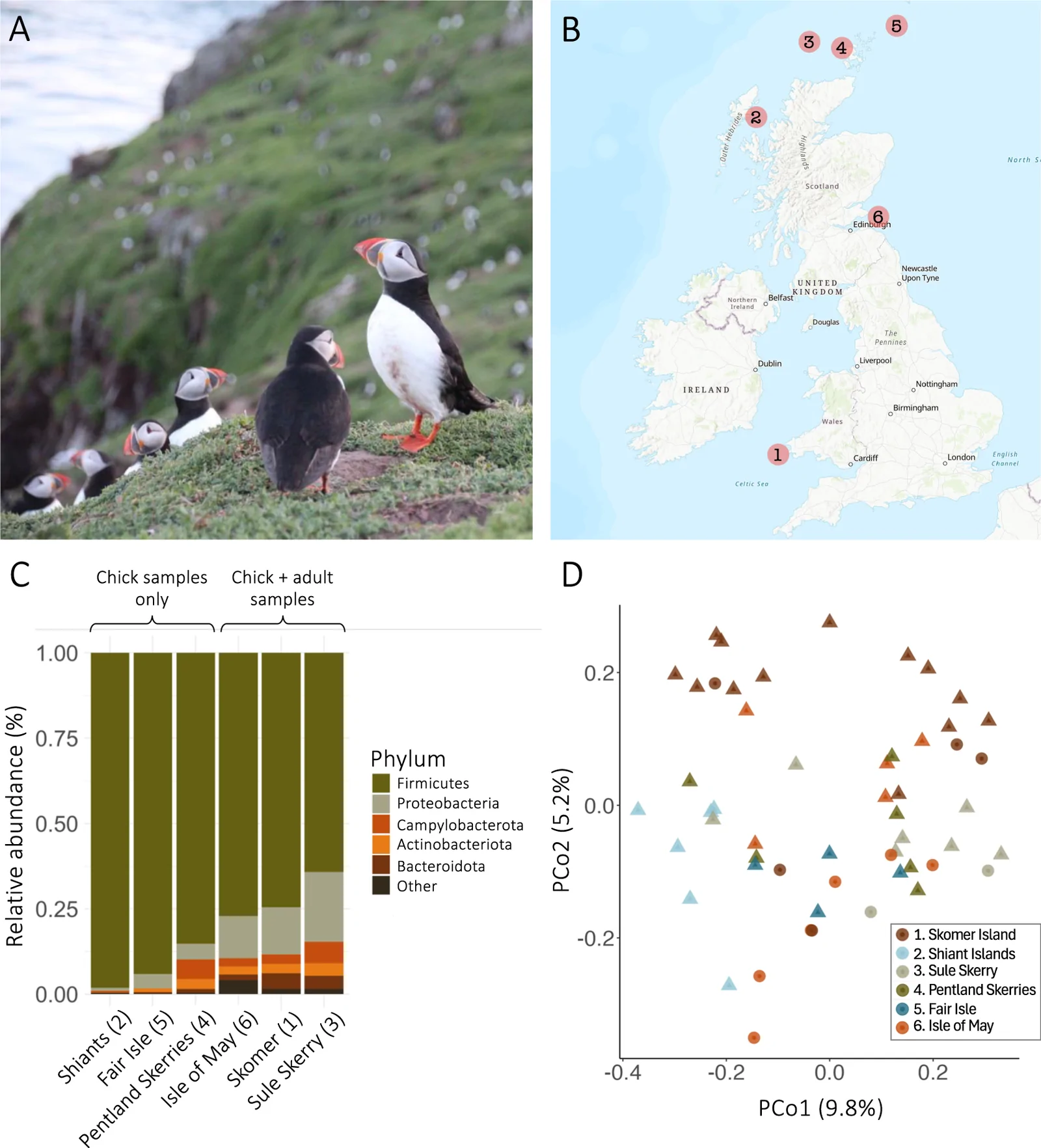
(A) Atlantic puffin (*Fratercula arctica*) (photo: Eveliina Hanski). (B) Map showing geographic locations of six British Atlantic puffin populations included in the study: (1) Skomer Island, (2) Shiant Isles, (3) Sule Skerry, (4) Pentland Skerries, (5) Fair Isle and (6) Isle of May. (C) Mean relative abundance of bacterial phyla across six populations, ordered by decreasing relative abundance of Firmicutes, the dominant phylum. Rare taxa (mean relative abundance less than 5% and prevalence less than 5% across samples) are under ‘Other’. (D) Principal coordinate analysis of adult (*n* = 13) and chick (*n =* 41) puffins on Jaccard distance. Microbial community similarity between sample pairs increases with point proximity. Shape indicates age category; *circle =* adult, *triangle =* chick.

A total of 153 faecal samples were collected in July 2021 from seven British populations; Skomer Island (Pembrokeshire, Wales), Skokholm Island (Pembrokeshire, Wales), Fair Isle (Shetland Islands, Scotland), Shiant Isles (Hebrides, Scotland), Sule Skerry (Orkney Islands, Scotland), Pentland Skerries (Orkney Islands, Scotland) and Isle of May (Firth of Forth, Scotland) (table 1; see map of islands for which samples were successfully sequenced in figure 1B). Samples were collected from adults and chicks. Chicks were estimated to be approximately two–five weeks old at the time of sampling, still residing in their burrows and dependent on parental provisioning. Adults were identified as breeding individuals only, without further age determination, and sex was indeterminate for both adults and chicks. Nest ID was not recorded for sampled individuals, and therefore we could not account for potential shared nest origin among samples from the same population. Samples were collected opportunistically during routine monitoring of nests or annual ringing campaigns by briefly holding the bird over a sterilized sampling dish for up to 1 minute. As puffins commonly defecate upon capture, samples were obtained when possible within this period. If no sample was produced, individuals were processed according to standard protocols or released, ensuring minimal additional handling time. All sample collection was conducted by field personnel licensed for bird handling under UK regulations. Faecal samples were collected from a sterile sampling dish using a sterilized spatula. Samples were preserved in DNA/RNA Shield (Zymo Research, USA), shipped to the laboratory at ambient temperature, and stored at −80°C until DNA extraction (five months).

**Table 1.** Gut microbiota profiles were successfully constructed for 54 samples from six British Atlantic puffin populations.

	adult				chick			
population	number of successful samples (failed)	read count range (mean)	asymptotic ASV richness range (mean)	asymptotic Shannon diversity range (mean)	number of successful samples (failed)	read count range (mean)	asymptotic ASV richness range (mean)	asymptotic Shannon diversity range (mean)
Fair Isle	0 (0)	N/A	N/A	N/A	4 (11)	75 370–118 312 (101 321)	47.2–265 (128)	1.52–12.8 (4.59)
Isle of May	6 (1)	5491–75 977 (46 910)	11–223 (95)	1.36–134.0 (23.9)	5 (5)	14 270–138 041 (60 900)	60–174 (121)	4.99–20.8 (11.7)
Pentland Skerries	0 (6)	N/A	N/A	N/A	6 (8)	24 851–142 664 (69 666)	47–276 (122)	3.33–13.8 (6.45)
Shiants	0 (1)	N/A	N/A	N/A	6 (10)	7094–121 209 (66 755)	17–58.1 (36.9)	1.73–9.36 (4.70)
Skomer	5 (20)	5748–198 796 (49 789)	38–260 (127)	2.66–92.1 (39.9)	13 (15)	14 038–135 838 (75 343)	30–281 (123)	5.45–50.1 (12.1)
Sule Skerry	2 (9)	5447–27 778 (16 612)	101–152 (127)	3.39–84.6 (44.0)	7 (9)	23 076–110 602 (71 731)	44–221 (145)	1.78–13.1 (7.53)
Skokholm	0 (4)	N/A	N/A	N/A	0 (0)	N/A	N/A	N/A

*Note:* The table reports the number of successfully processed samples for adults and chicks separately, with the number of failed samples shown in brackets. For each group, the range and mean of sequencing read counts are reported, along with asymptotic estimates of amplicon sequence variant (ASV) richness and Shannon diversity derived from iNEXT (see §2). These diversity estimates account for variation in sequencing depth and are used in the alpha diversity comparisons reported in the main text.

### 2.2. Laboratory work

Faecal samples were randomized into eight batches of up to 23 samples for DNA extraction. DNA was extracted using the ZymoBIOMICS DNA MiniPrep kit (spin-column format) according to the manufacturer's instructions (Zymo Research, USA). A negative control (DNAse-free H_2_O) was included in each DNA extraction batch at a varying position to account for potential systematic cross-contamination between positions. Microbiota profiling was conducted using amplicon sequencing, targeting the V4–V5 regions of the 16S rRNA gene using primers 515F and 926R [41,42]. Library preparation was conducted in two 96-well plates of up to 94 samples (comprising true samples as well as negative controls). Each library preparation also included a negative polymerase chain reaction (PCR) control (at a varying position). Amplicons were sequenced in a single sequencing run using the Illumina MiSeq platform (Reagent kit v. 3, 2x300 bp chemistry). Library preparation and sequencing were conducted at the Integrated Microbiome Resource, using the protocol described in [43]. All negative controls for DNA extraction and PCR reactions were sequenced intermixed with true samples, alongside the sequencing facility's negative reaction for the amplicon sequencing.

### 2.3. Data processing

Data processing and subsequent analysis were conducted using R v. 4.1.2. Demultiplexed sequencing reads were processed through the *DADA2* pipeline [44]. Taxonomy was assigned using the SILVA rRNA database v. 138.1 [45]. Packages *DECIPHER* [46] and *phangorn* [47] were used to build a phylogenetic tree. A total of eight amplicon sequence variants (ASVs) were detected across all negative DNA extraction and library preparation controls (*n* = 9), with a maximum read count of 77 for any given control (range 0–77, mean 15; for comparison, mean read depth was 66176 for true samples). Using these negative controls, we tested for the presence of any potential contaminants using the R package *decontam* [48] using the prevalence method, in which the prevalence of each sequence in biological samples is compared with that in negative controls (conducted separately with extraction and library preparation controls). A sequence was considered a contaminant if it reached a probability of 0.1 in Fisher's exact test used in *decontam*. Across the two tests (decontam with either extraction or library preparation controls), one ASV contaminant was identified and filtered from the data before subsequent analysis.

Samples with fewer than 100 reads were first removed as sequencing failures (*n* = 72), reducing the dataset from 153 to 81 samples. The package *iNEXT* [49] was then used to generate sample completeness and rarefaction curves at both ASV and genus level. *iNEXT* estimates sample completeness as the probability that the next sequenced read represents a previously observed taxon, providing a coverage-based criterion for sequencing sufficiency. These curves at both taxonomic levels supported a sequencing depth threshold of 5000 reads, below which a substantial proportion of samples remained incomplete. Samples below this threshold (*n* = 25) were removed. Asymptotic diversity estimates were calculated using *iNEXT* as Hill numbers for ASV richness (*q* = 0) and Shannon diversity (*q* = 1), after which ASVs that were never observed at a count greater than 1 in any individual sample were removed from the dataset (*n* = 245) to reduce potential residual sequencing artefacts. The package *phyloseq* [50] was used to normalize ASV counts to proportional abundance. Finally, two duplicates (two faecal depositions were sampled twice) were removed from the data. After data processing, 54 unique samples from six populations were retained for analysis (table 1) with a mean read depth of 66176 (range 5447–198796). Across these samples, 2096 ASVs were detected, with each sample having 113 ASVs on average (range 11–277). Both adult and chick samples were available for analysis in three of the six populations (Skomer, Isle of May and Sule Skerry; table 1).

### 2.4. Phenotypic profiles of bacteria

Bacterial aerotolerance was determined using a custom database as described in [17]. Briefly, bacterial genera were categorized into ‘obligate anaerobes’ (when specifically listed as *obligate* anaerobes) and ‘aerotolerants’ (for all facultative anaerobes, microaerophiles, facultative aerobes and aerobes) or ‘unknown’ (when information was not available) using Bergey's Manual of Systematics of Archaea and Bacteria or additional peer-reviewed references when information was not available in the manual [51]. As aerotolerance was assigned at the genus level, intrageneric variation in some taxa may introduce misclassification in a minority of genera; this is an inherent limitation of genus-level taxonomic resolution. As this classification was applied uniformly across all samples using a consistent reference database, any resulting misclassification error should not systematically bias comparisons between age groups or populations. The aerotolerance data used here are available online (see Data accessibility).

### 2.5. Data analysis

To investigate the proportion of gut microbiota variation explained by population and age (adult vs chick), we performed marginal permutational multivariate analyses of variance (PERMANOVA) on Jaccard and Aitchison distances using the adonis2 function in the R package *vegan* [52]. Jaccard distance was calculated using the distance function in package *phyloseq,* with the binary argument set to TRUE, such that relative abundances of taxa are not considered. Aitchison distance was calculated by first applying a centred log-ratio (CLR) transformation using the transform function from the package *microbiome* [53]. This CLR transformation replaces exact zero relative abundances with a pseudocount (min(relative abundance)/2) before taking logarithms. The distance function from the package *phyloseq* was then used with the method set to euclidian. DNA extraction batch and read depth were included as predictors. Beta dispersion by population ID and age category was tested for using the betadisper function in package *vegan* [52], a method that is sensitive to unbalanced group sizes.

To study predictors of gut microbial similarity between individuals, we used a dyadic Bayesian regression framework (function brm) with the R package *brms* [54]. It comprises a pairwise generalized linear mixed model predicting gut microbial similarity (on Jaccard distance, measured as described above) with other measures of a sample pair (e.g. spatial proximity). We ensured that similarity scores (Jaccard indices) fell strictly within the (0,1) interval by applying a standard transformation: (*x* × (*n* − 1) + 0.5)/*n*, where *n* is the sample size. We then used logit link functions of family *beta*. The effect of spatial proximity on microbiota similarity was estimated separately for (i) *adult–adult* and (ii) *chick–chick* sample pairs with two models. As the sample size for adults was substantially smaller than that of chicks (table 1), we repeated the chick model using a subset of randomly selected sample pairs matched in population representation and sample size to the adult dataset, with the only exception that the Isle of May had five instead of six samples (table 1). Models used population ID similarity (same vs different population) and at-sea proximity (puffins do not fly over land, so at-sea distance represents a more ecologically relevant distance between populations than absolute distance) as predictors. For this, at-sea distance was scaled to the (0,1) range as described above and converted to proximity (1 − normalized distance) for more intuitive interpretation. These spatial analyses were repeated on the anaerobic and aerobic communities of the microbiota (as aerobic taxa predominantly spread through environmental exposure while anaerobic taxa spread through social transmission [18]), with differences in the relative abundance of taxa with unknown aerotolerance included as a fixed effect.

For the investigation of microbiota variation across age categories, we used two approaches. First, we predicted microbial similarity with pairwise age category similarity (same vs different age category). Second, to estimate whether beta diversity varies across the two age categories, we predicted microbial similarity with pairwise age category (*adult–adult* or *chick–chick*; only sample pairs with the same age category were included). Both models investigating age effects on microbial similarity included population ID similarity as a covariate. For identifying taxa driving age effects in the microbiota, we used a ‘drop-one-taxon-out’ approach. In each iteration, a single genus was excluded, and all ASVs assigned to that genus were removed from the dataset before re-measuring the Jaccard index and running the brm model as described above (Jaccard distance ∼ pairwise age category + …). This was repeated until every genus, along with all of its associated ASVs, had been excluded once. We then quantified ‘importance’ scores for each of the 465 genera, as described in [18], reflecting the extent to which excluding a genus from the data reduced the certainty of the age effect estimate (i.e. increased the credible interval width).

All dyadic beta regression models included DNA extraction batch similarity (same vs different extraction batch) as well as read depth difference of the sample pair as fixed effects. To account for the inherent autocorrelation of pairwise values, all dyadic models included a multi-membership random effect (1|mm(ID1, ID2) [18].

To assess whether alpha diversity varied across age categories, we fitted two separate models for asymptotic Shannon diversity and asymptotic ASV richness (estimated with *iNEXT* [49], see details in §2.3) using *brms* [54]. For Shannon diversity, we used a *zero-inflated beta* regression model, with a precision (phi) term predicted by age to account for potential differences in dispersion. The response variable was scaled to the (0,1) range. For ASV richness, we fitted a *Gaussian regression model* with a log-transformed response. In both models, age category (adult vs chick) and read count were included as fixed effects, with population ID as a random effect to account for population-level variation. Alpha diversity models were run with (i) all samples and (ii) all except the Shiants samples, since observed ASV richness was substantially lower in the Shiants population (table 1).

To examine whether microbiota aerotolerance varied by age (anaerobe to aerobe ratios have been observed to increase with age in several mammalian species [14,17,55]), we similarly implemented a brm model. The proportion of aerotolerant taxa out of taxa with known aerotolerance was used as the response variable. Age category, scaled read count, DNA extraction batch and summed relative abundance of taxa with unknown aerotolerance were included as fixed effects, while population ID was included as a random effect to account for variation across populations. Read count was scaled to improve model convergence and ensure comparability across samples. Since the response variable included values of exactly 1, we specified a *zero-inflated beta* distribution to appropriately model the data.

For all brm models, posterior checks were conducted to confirm reliable model performances. Here, we ensured that the chains had converged, Rhat values were less than 1.05, bulk effective sample sizes at least 100 times the number of chains and no smaller than 10% of total posterior draws, and that the sampler took small enough steps to avoid excess (greater than 10) divergent transitions after warm-up. In addition, posterior predictive checks were performed to assess model fit, confirming that the simulated posterior distributions closely matched the observed data, with only minor deviations in certain regions.

Networks of pairwise microbiota similarity were constructed to visualize similarity between puffin populations based on chick samples. Only chick samples were included, as adult samples were available for only three of the six populations (table 1). Networks were generated separately for the whole microbiota, anaerobic taxa and aerobic taxa (aerotolerance determined at the genus level, see §2.4). To ensure comparability across populations with uneven sample sizes, networks were based on 100 iterations of random subsampling of four chick samples per population (the sample size available in the population with the fewest chick samples; table 1). In each iteration, presence–absence data were used to calculate ASV richness within populations and Jaccard similarity (*n* ASVs present in both populations / total *n* ASVs across the two populations) between population pairs. Final node sizes and edge thicknesses represent the average ASV richness and Jaccard index, respectively, across all iterations.

## 3. Results

### 3.1. Atlantic puffin gut microbiota composition and structure across six British populations

**Figure 2.**
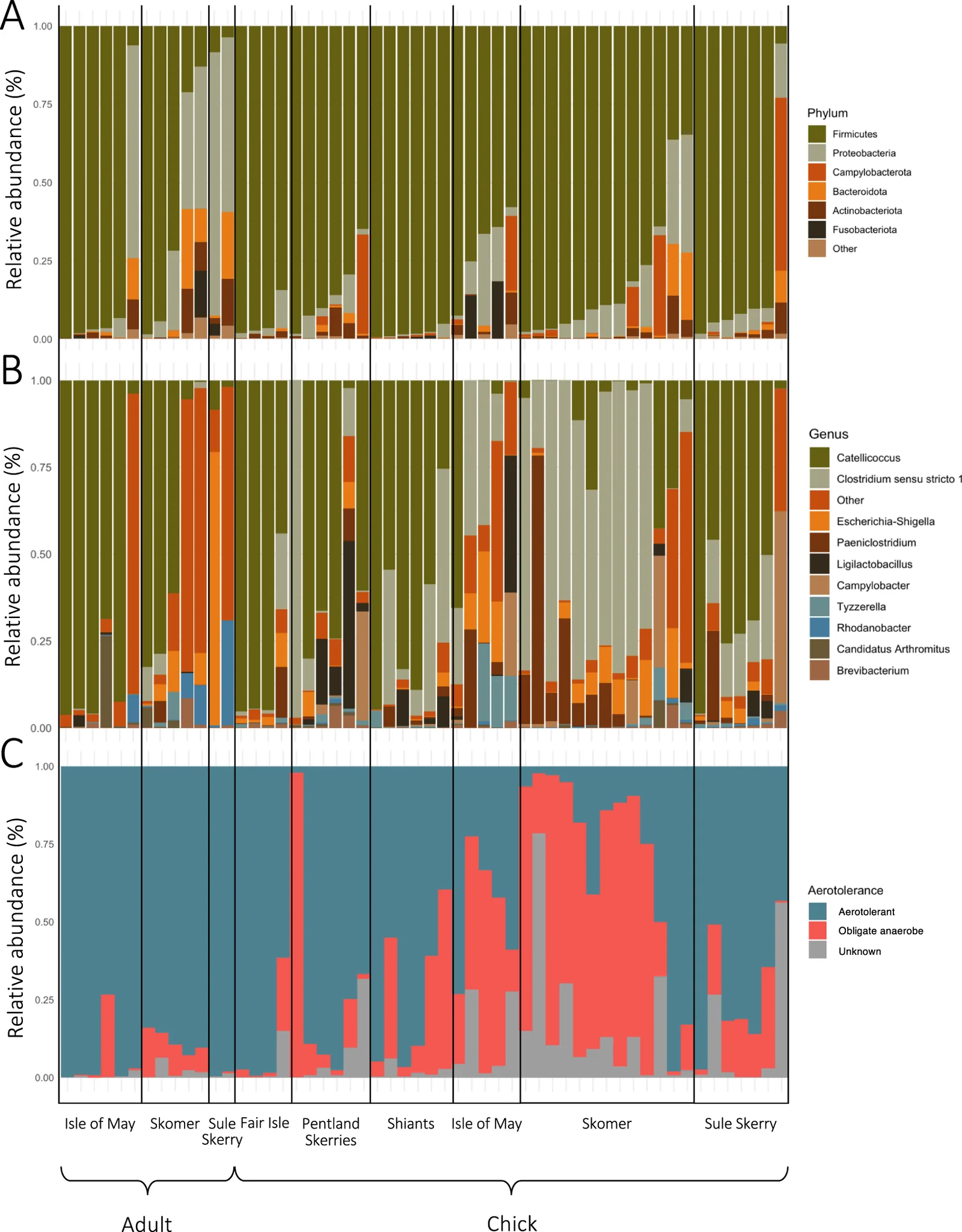
Gut microbiota composition and oxygen tolerance of individual Atlantic puffins. Each vertical bar represents one puffin sample (*n* = 54). Samples are grouped by age category (adult vs chick) and within age category, ordered by population (Isle of May, Skomer, Sule Skerry, Fair Isle, Pentland Skerries, Shiants). Within each population–age group, samples are ordered by decreasing relative abundance of the dominant phylum, Firmicutes. (A) Relative abundance of bacterial phyla across samples. Taxa with mean relative abundance less than 5% and prevalence less than 5% are grouped under ‘Other’. (B) Relative abundance of bacterial genera across samples. Rare genera (mean relative abundance less than 5% and prevalence less than 5%) are grouped under ‘Other’. (C) Relative abundance of aerotolerant, obligate anaerobic and bacteria of unknown aerotolerance across samples. Genera with ambiguous or mixed aerotolerance classifications are grouped as ‘Unknown’.

We profiled the bacterial component of the Atlantic puffin gut microbiota in adults (*n* = 13) and chicks (*n* = 41) from six populations in the United Kingdom. At the phylum level, the puffin gut microbiota was dominated by Firmicutes (mean relative abundance 78.6%) and Proteobacteria (11.5%), followed by Campylobacterota (3.1%), Bacteroidota (2.4%) and Actinobacteriota (2.4%) (figures 1C and 2A). The most common genera were *Catellicoccus* (43.1%), *Clostridium sensu stricto 1* (22.2%)*, Escherichia–Shigella* (5.3%) and *Paeniclostridium* (5.0%) (figure 2A; supplementary material, figure S1B).

Both population and age category (adult vs chick) significantly explained variation in microbiota composition, though age had markedly lower explanatory power than population. Population explained 15.6% of variation in the taxa detected in the samples (marginal PERMANOVA; binary Jaccard index, *population*: *R*^2^ = 0.156, *F* = 1.838, *p* = 0.001; *age*: *R*^2^ = 0.023, *F* = 1.337, *p* = 0.032; beta dispersion significant for *population*: *F* = 4.511, *p* = 0.005, but not *age*: *F* = 2.997, *p* = 0.084; figure 1D) and 13.6% of variation in the relative abundances of these taxa (Aitchison distance, *population*: *R*^2^ = 0.136, *F* = 1.661, *p* = 0.001; *age*: *R*^2^ = 0.030, *F* = 1.788, *p* = 0.008; beta dispersion, *population*: *F* = 2.223, *p* = 0.073; *age*: *F* = 1.766, *p* = 0.191; supplementary material, figure S1B).

Overall, the puffin gut microbiota was dominated by aerotolerant taxa (mean relative abundance 70.4%, s.d. 29.3%; mean relative abundance of anaerobic taxa 31.5%, s.d. 34.0%; figure 2C; supplementary material, figure S1C; aerotolerance was determined at genus level, see §2).

### 3.2. Age-related variation in microbial diversity and composition

**Figure 3.**
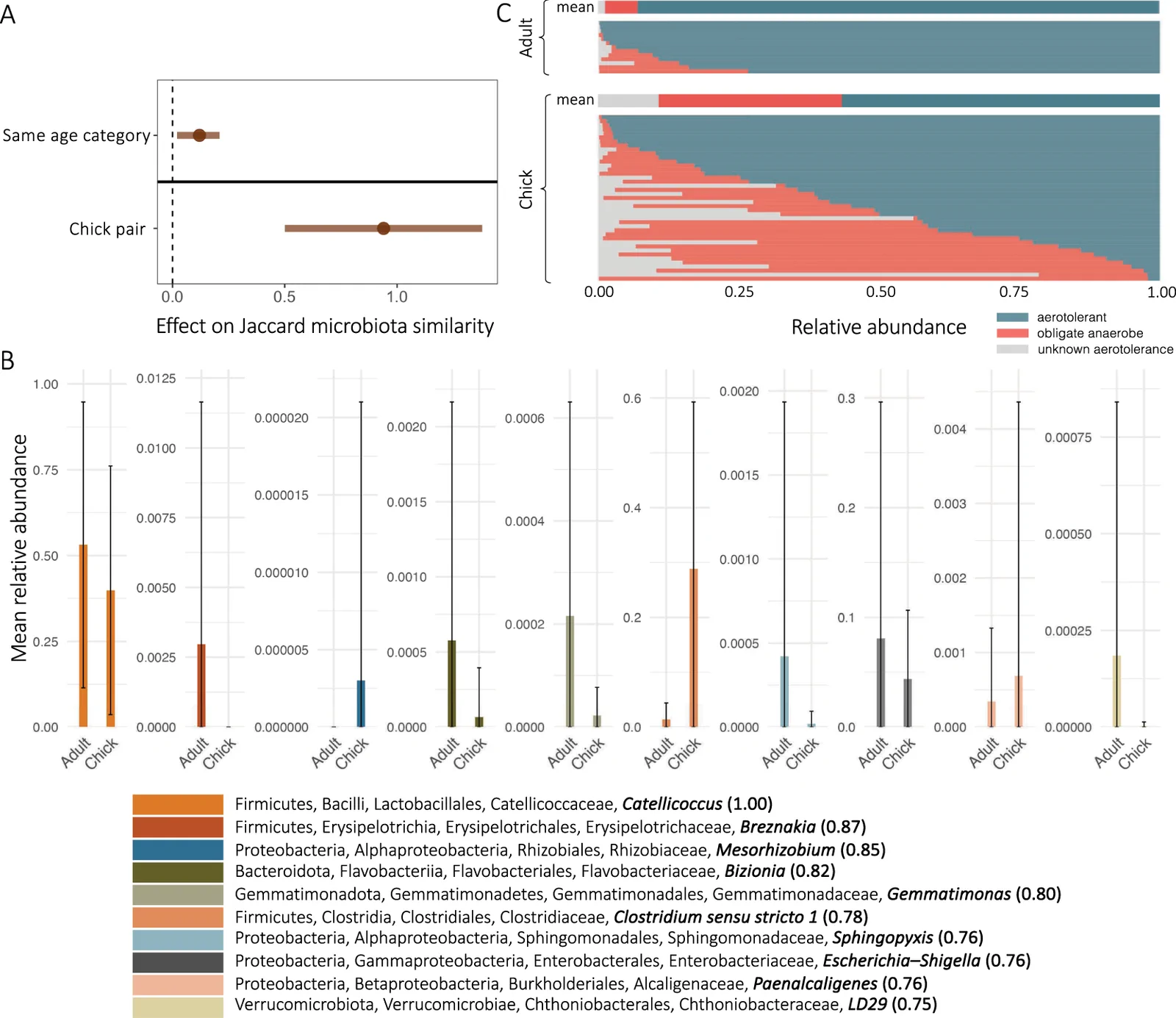
(A) Estimated effects of age similarity (*top*) and category (*bottom*) on pairwise gut microbiota similarity (Jaccard distance), based on two Bayesian regression models (brm). The top model (‘same age category’) estimates the effect of whether two samples are from the same versus different age categories (reference: different age). The bottom model (‘chick pair’) estimates the effect of being a chick pair versus an adult pair, among within-age comparisons (reference: adult pair). Points show posterior means; horizontal lines show 95% credible intervals (CIs). A variable significantly predicts community similarity if its CIs does not overlap zero. The effect sizes of the estimates between the two models are not directly comparable. Y-axis labels (‘Same age category’, ‘Chick pair’) indicate the predictor levels being compared with their respective reference groups. (B) Mean relative abundance (± s.d.) of the top 10 bacterial genera contributing most strongly to age-related differences in microbiota composition, identified using a ‘drop-one-taxon-out’ approach (see §2). Importance scores (in parentheses) reflect the influence of each genus on the certainty of the age effect estimate (i.e. the extent to which its removal widens the CI): high importance scores therefore reflect consistent age-associated occurrence and need not correspond to high mean relative abundance. (C) Stacked bar plots showing the aerotolerance profile of gut microbiota across individual puffins, grouped by age. Each thin bar represents an individual, ordered by the relative abundance of aerotolerant taxa within the age category. The wider bars at the top of each cluster show the mean relative abundances for adults (top) and chicks (middle). Colours indicate bacterial oxygen tolerance category: aerotolerant (*blue*), obligate anaerobe (*red*) and unknown aerotolerance (*grey*).

Microbiota composition differed significantly between adults and chicks. Sample pairs from the same age category were more similar than those from different age categories (Bayesian regression model (brm): using *different* as the reference category, posterior mean for *same* was 0.12, 95% credible intervals (CIs) 0.02–0.21; see details for all models in §2). Among same-age pairs, chick microbiotas were more similar to one another than adult microbiotas (posterior mean for *chick–chick* pairs = 0.94, CIs 0.50–1.38), suggesting beta diversity (interindividual variation in microbiota composition) increased with age (figure 3A; see also individual-level phylum and genus profiles in figure 2).

In terms of alpha diversity, Shannon diversity was significantly lower in chicks compared with adults (posterior mean for *chick* = −1.99, CIs −2.85 to −1.13), and also more consistent (i.e. lower variability across individuals; posterior mean *phi* = 4.06, CIs 3.27–4.86). However, ASV richness did not differ significantly between age groups (posterior mean for *chick* = 0.03, CIs −0.10 to 0.17). To assess whether the relatively low ASV richness in the Shiants population (mean = 36.9, compared with 95–145 in other populations; table 1) may have influenced these patterns, we repeated the models excluding this population. Results were nearly identical: Shannon diversity remained significantly lower in chicks (posterior mean for *chick* = −1.94, CIs −2.80 to −1.08), while ASV richness still showed no significant age-related difference (posterior mean for *chick* = 0.06, CIs −0.08 to 0.19).

To further characterize age-related differences in the puffin microbiota, we next examined taxonomic composition. Overall, the relative abundance of Proteobacterial taxa was higher in adults than in chicks (figure 2A), while at the genus level, chicks had higher abundances of *Clostridium sensu stricto 1*, which was also among the top 10 genera with the highest importance scores in driving the age effect on the puffin microbiota (figure 3B; supplementary material, table S1 and figure S1B). These genera were taxonomically diverse, spanning five distinct phyla: Bacteroidota, Firmicutes, Proteobacteria, Gemmatimonadota, and Verrucomicrobiota. This approach assesses how much each taxon contributes to age-related microbiota differences by systematically removing them from the data and measuring the change in model certainty. While several of these taxa had low mean relative abundances (less than 0.2%) in both adult and chick samples, importance scores reflect the consistency of age-associated occurrence rather than absolute abundance: a taxon that is reliably present in one age category and absent in the other will strongly influence the age effect estimate regardless of its mean relative abundance. Others, such as *Catellicoccus* (approx. 53% in adults, approx. 40% in chicks) and *Escherichia–Shigella* (approx. 8% in adults, approx. 4% in chicks), were substantially more abundant on average, although their relative abundances varied widely across individuals within each age category (figures 2B and 3B; supplementary material, table S1). This compositional divergence extended to microbial oxygen tolerance: on average, chicks had a significantly lower proportion of aerotolerant bacteria than adults (posterior mean for *chick* = −1.81, CIs −2.79 to −0.81; figures 2C and 3C; supplementary material, figure S1C).

### 3.3. Puffin chicks but not adults show spatially structured gut microbiota shaped by oxygen tolerance

Spatial effects on microbiota similarity were strongly age dependent. In adults, neither population similarity (same vs different population) nor at-sea proximity significantly predicted microbiota similarity (posterior mean for *same population* = 0.43, CIs −0.44 to 1.30; posterior mean for *at-sea proximity* = −0.38, CIs −1.30 to 0.53; see at-sea population distances in supplementary material, figure S2). In chicks, however, microbiota similarity was significantly higher in sample pairs from the same rather than different populations, while at-sea proximity had no significant effect (posterior mean for *same population* = 0.79, CIs 0.62–0.96; posterior mean for *at-sea proximity* = −0.08, CIs −0.28 to 0.13). These effects were maintained in a reduced dataset matched to the adult sample structure, though the population effect was weaker and only marginally significant (posterior mean for *same population* = 0.60, CIs 0.03–1.19; posterior mean for *at-sea proximity* = −0.20, CIs −0.81 to 0.41; table 1).

**Figure 4.**
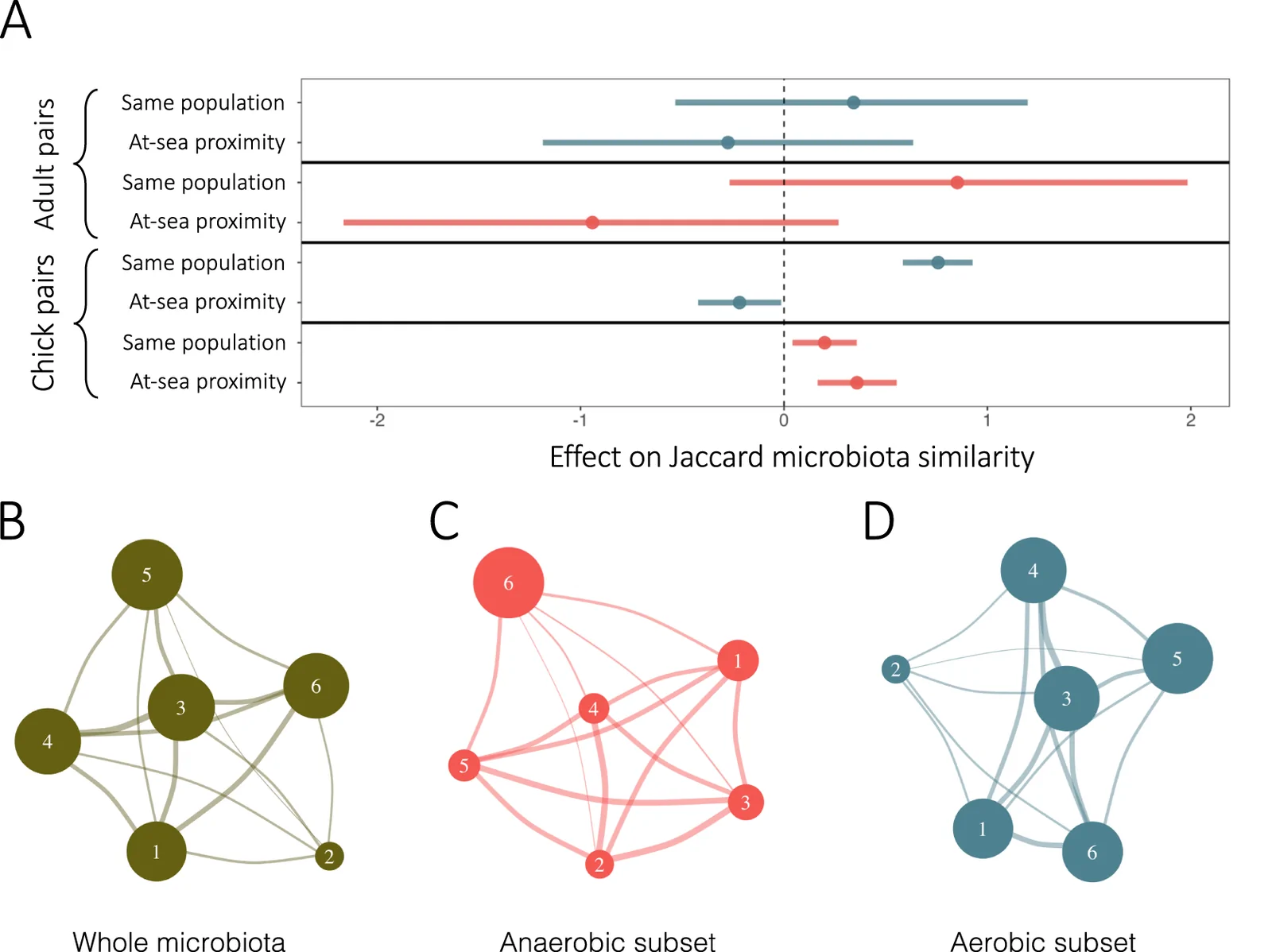
(A) Estimated spatial effects on pairwise gut microbial similarity on Jaccard distance in adult and chick sample pairs from Bayesian regression models (brm) that predicted pairwise similarity of aerobic (*blue*) or anaerobic (*red*) microbiota communities in adult–adult or chick–chick sample pairs with population ID similarity (same vs different population with ‘different population’ as reference category) and at-sea proximity (scaled to 0–1 for easier interpretation; sample sizes are given as the number of unique pairwise comparisons: adult–adult *n =* 78 dyads; chick–chick *n* = 820 dyads). Points are posterior means, and lines are 95% CIs. A variable significantly predicts community similarity if its CI does not overlap zero, and effects are significantly different if their CIs do not overlap. Black horizontal lines separate different models. Effect sizes are model-specific and not directly comparable across models. (B–D) ASV sharing networks between chick samples from puffin populations (1. Skomer, 2. Shiant Isles, 3. Sule Skerry, 4. Pentland Skerries, 5. Fair Isle, and 6. Isle of May) for the whole microbiota (B), anaerobic microbiota (C) and aerobic microbiota (D). Nodes represent puffin populations, with node size proportional to the average ASV richness per population based on 100 iterations of randomly sampled sets of four chick samples. Edges represent pairwise microbiota similarity (not direct ASV sharing or transmission) between populations, quantified as the mean Jaccard index across the same iterations. Edge thickness is scaled to average Jaccard index values. Edge length is determined by a force-directed layout and reflects overall network structure, but does not correspond to spatial distance or microbiota similarity.

When splitting the microbiota into anaerobic and aerobic components, additional spatial patterns emerged. In chicks, population similarity and spatial proximity both predicted anaerobic microbiota similarity (posterior mean for *same population* = 0.20, CIs 0.04–0.36; posterior mean for *at-sea proximity* = 0.36, CIs 0.16–0.55), while aerobic microbiota were more similar within populations but became less similar between populations that were geographically closer (posterior mean for *same population* = 0.76, CIs 0.58–0.93; posterior mean for *at-sea proximity* = −0.22, CIs −0.42 to −0.01; figure 4A). Although this latter effect was only marginally significant, it suggests both microbial groups show spatial structuring beyond population similarity. However, these opposing spatial patterns may effectively cancel each other out in whole-community analyses, obscuring broader spatial trends (figure 4A). In adults, by contrast, neither anaerobic nor aerobic microbiota showed significant spatial structuring (figure 4A).

To better understand microbiota differentiation between puffin populations, we visualized networks of pairwise microbiota similarity. Networks were constructed for the whole microbiota (figure 4B) as well as for anaerobic and aerobic communities separately (figure 3C,D). The full network revealed strong similarity among most populations but also highlighted reduced overlap with the Shiant Isles (node 2), suggesting some population-specific distinctiveness. When partitioned by oxygen tolerance, these patterns diverged. Anaerobic microbiota exhibited broadly connected and relatively even distribution across populations (indicated by the relatively uniform edge thicknesses between nodes). By contrast, the aerobic microbiota exhibited a pronounced separation of the Shiant Isles, with fewer common ASVs than other populations. This suggests that the observed negative effect of spatial proximity on aerobic microbiota similarity (figure 4A) may be largely driven by this single, distinct population rather than a consistent pattern across all populations.

## 4. Discussion

Birds are key contributors to ecosystem function [56]. Yet, many of the host traits supporting these benefits are tightly linked to the gut microbiota [57], a component of avian biology that remains underexplored. Using samples from six Atlantic puffin populations from the British Isles, we demonstrate that microbiota composition and structuring is strongly age dependent and displays patterns that suggest divergence from mammalian models.

Overall, the puffin gut microbiota was dominated by Firmicutes and Proteobacteria, with relatively low levels of Bacteroidota. This composition resembles that of other seabirds such as shearwaters [4], fulmars [4] and thick-billed murres [5], as well as Seychelles’ warblers [10] and bats [58,59], and is broadly consistent with prior observations from UK puffins that found Proteobacteria and Firmicutes among the dominant phyla [60]. By contrast, gut communities in non-volant terrestrial mammals are typically dominated by Firmicutes and Bacteroidota [14,17,61,62], while marine mammals show mixed patterns [63–66], suggesting that marine ecology alone does not consistently drive high Proteobacteria abundance.

Age-related microbiota dynamics in birds are of particular interest owing to their distinct developmental context, probably shaped by environmental exposure rather than the primary maternal transmission routes (birth canal and lactation) that influence mammalian microbiota [14,17,67,68]. This may result in more variable early-life microbial dynamics across individuals, populations, and bird species, in contrast to the relatively conserved trajectories seen in several mammalian species [14,17,69]. Our findings support this view, revealing clear age-related shifts in gut microbiota diversity and composition.

Shannon diversity was significantly higher in adults than in chicks (who were approx. two–five weeks old at sampling), while ASV richness remained comparable, suggesting that evenness, rather than richness, increases with age. This pattern diverges from mammals, where both richness and evenness typically increase during early development [12–14,17], but aligns with more heterogenous age dynamics reported in other bird species [21,22,34,35,70,71]. The increased evenness (reflected in higher Shannon diversity) in adults may reflect dietary diversification and immune system maturation, which could promote more even microbial communities. One possible explanation for the lack of richness differences is that since chick samples were collected weeks after hatching, initial colonization dynamics may have already been stabilized (microbiota diversity is known to change rapidly over a matter of days during early chick development in great tits [22] and ostrichs [35]), limiting our ability to capture early richness changes.

These age-related diversity patterns were accompanied by marked compositional differences between chicks and adults, driven primarily by *Catellicoccus*, which was the most abundant genus in our dataset and showed significantly higher relative abundances in adults. The dominance of *Catellicoccus* in puffins has been reported previously [60] and is also observed in gulls [72] and thick-billed murres [5], with lower-level detection in waterfowl [73] and waders [74]. The only described species, *Catellicoccus marimammalium*, exhibits features of a host-adapted lifestyle, including limited biosynthetic capacity and specialized nutrient transport systems [75], and has been associated with longer telomere length in yellow-legged gulls [76], hinting at potential links to host health. Originally isolated from porpoise and grey seal [77], *Catellicoccus* has been primarily reported in seabirds and marine mammals, and appears to be uncommon in terrestrial mammals. This distribution pattern suggests that shared habitat (rather than host phylogeny alone) may influence its prevalence in marine-associated mammals. In puffins, its higher relative abundance in adults could reflect increased marine exposure. Given its dominance in multiple avian taxa, signs of host adaptation, and age-association across species, *Catellicoccus* may represent a ‘core’ member of the avian gut microbiota with functional importance.

Interestingly, we found that the overall composition of gut microbiota became increasingly individualized with age. Chick microbiotas were more similar to one another than those of adults, suggesting that microbial communities diverge between individuals as they grow older. This pattern is consistent with findings in some other bird species [22] but contrasts with patterns observed in common mammalian systems (primates and mice), where early-life microbiotas tend to be more variable between individuals and converge over time [13,14,17]. The greater similarity in chick microbiotas may reflect their narrow age range and more constrained environments compared with adults: chicks were all sampled within a narrow age range (approx. two–five weeks), whereas adults probably spanned a much wider age range (puffin breeding age can range approx. 5–40 years). Furthermore, adults also forage widely [78,79] and migrate across large marine areas throughout the year, with overlap between the non-breeding distribution of different populations but substantial individual differences [38], potentially encountering similar environmental microbes regardless of their breeding population, which may reduce population-level differences while still increasing variation between individuals.

Indeed, spatial structuring of the microbiota was evident in chicks but not adults. Chick microbiotas were more similar within than between populations, consistent with local environmental shaping, while adult microbiotas showed no such geographic structure. This age-dependent spatial pattern supports the hypothesis that avian microbiota assembly is more environmentally driven than in mammalian species. Puffin chicks remain in underground burrows for several weeks post-hatching [36] and are therefore exposed to locally distinct soil microbial communities. These early-life exposures probably shape the initial microbial community, contributing to the population-level signal observed. In early life, microbial communities are also thought to be under weaker host physiological control [55,80,81], making them more susceptible to local environmental influences. As hosts mature, increased immune modulation and physiological filtering may contribute to greater host-driven divergence, as well as a weakening of geographic signals. In puffins, this may help explain why adult microbiotas are both more variable across individuals (i.e. higher beta diversity) and less spatially structured. The absence of spatial structure in adults may also partly reflect our smaller adult sample size, though when chick analyses were restricted to match the adult sample structure, a population effect was still marginally detectable.

To further understand the ecological drivers shaping puffin gut microbial communities, we partitioned the microbiota by oxygen tolerance. On average, chicks harboured a higher proportion of obligate anaerobes, whereas adults were dominated by aerotolerant taxa. This contrasts patterns observed in mammals [17,55,68,82] and may be explained by a combination of ecological, physiological and/or behavioural factors unique to puffins or potentially seabirds more broadly. For instance, adult puffins spend significant time foraging at or near the ocean surface, where they are exposed to bacteria that are probably aerotolerant owing to the high oxygen levels in surface seawater. Although chicks are similarly exposed to soil microbes, which are expected to be aerobic, their exposure may well be more passive, as they are unlikely to actively ingest soil in the way adults may take in seawater while feeding. Further research is needed to determine whether the observed pattern in the ratio of anaerobes to aerobes is common in seabirds and what factors (e.g. immune modulation, feeding frequency) might be driving it. It is also important to note that in our cross-sectional microbiota profiles, we cannot distinguish whether detected microbes—including the aerotolerant taxa in adults—represent resident members of the gut microbiota or transient taxa associated with recent environmental exposures, which could be resolved through longitudinal sampling.

The spatial structuring of the microbiota supports a divergence in how anaerobic and aerobic microbial communities respond to host and environmental factors. In chicks, anaerobic microbiotas were more similar within populations and between geographically proximate populations. By contrast, aerobic microbiota showed higher similarity within populations but decreased similarity with increasing spatial proximity. This negative spatial effect, while only marginally significant, may reflect environmental filtering or localized divergence in aerobes, potentially driven by a single distinct population. The Shiant Isles exhibited highly distinct aerobic microbiota and also had markedly lower ASV richness for both aerobic and anaerobic taxa, suggesting that reduced diversity and unique local conditions (such as island-specific geology or soil microbiota) may have contributed to its differentiation. These findings, consistent with a recent study of the Seychelles warbler in which anaerobic—but not aerotolerant—microbiota similarity was predicted by the closeness of cooperative breeding interactions [40], suggest that partitioning by oxygen tolerance may be a broadly informative approach for revealing microbial transmission dynamics that remain obscured in whole-community analyses of wild birds.

Despite yielding considerable advancements in our understanding of the Atlantic puffin gut microbiota, this study had limitations. Sequencing success was variable: 72 samples were removed prior to rarefaction analysis owing to extremely low read counts (less than 100 reads), probably reflecting failed DNA extractions or PCR inhibition [82,83]—a common challenge for avian microbiota studies [84] given the co-excretion of nitrogenous waste and gastrointestinal excrement together in the faeces [85]. A further 25 samples were removed for insufficient sequencing depth, resulting in small final sample sizes with uneven representation of populations and age categories. In particular, adult sample sizes were limited across all populations, and results from adult-specific comparisons (particularly Sule Skerry, where only two adult samples were available) should be interpreted with appropriate caution. In addition, genetic relatedness among chicks within populations is unknown, although gut microbiota heritability in wild animals is generally modest [80]. While our cross-sectional comparison of adults and chicks at a single time point reveals marked differences between the two age categories, longitudinal sampling from hatching through fledging would be needed to fully capture the microbiota assembly process and initial colonization dynamics in birds. Future studies would also benefit from investigating microbiota components beyond bacteria, such as fungi (the ‘mycobiota’), as well as exploring high-resolution functional changes in the puffin gut microbiota and their relationships to host health and fitness.

In conclusion, our study provides characterization of the gut microbiota in the Atlantic puffin, revealing a dynamic interplay between host age, microbiota composition, and spatial ecology. The patterns we document—including population-level structuring in chicks that disappears in adults, increasing individualization with age and increased aerotolerance—appear to contrast those observed in commonly used mammalian model systems. These findings highlight the need for further research into the ecological and evolutionary drivers of microbiota variation in birds and suggest that age-related microbiota dynamics may be more varied across vertebrates. As avian species face increasing environmental pressures, including global disease threats such as highly pathogenic avian influenza (HPAI), understanding factors driving variation in microbiota composition and what consequences microbiota development has on later health will be increasingly important. More broadly, our results contribute to a growing recognition that birds can serve as valuable comparative models for advancing microbiota research beyond the mammalian species.

## Data Availability

The 16S rRNA amplicon sequencing data used in this study have been deposited in the Sequence Read Archive (SRA) under accession code PRJNA1269590 (https://www.ncbi.nlm.nih.gov/bioproject/PRJNA1269590). Sample-specific metadata, aerotolerance data, taxonomy table, ASV relative abundance table as well as R code used in the study are publicly available at https://github.com/eveliinahanski/fratercula_arctica and have been archived within the Zenodo repository [86].
